# Chemical Composition, Antioxidant Potential, and Genotoxic Safety of Lamiaceae Essential Oils from Eastern Morocco: A Multimethod Evaluation

**DOI:** 10.3390/molecules31030400

**Published:** 2026-01-23

**Authors:** Abderrahman Makaoui, Abdelmonaem Talhaoui, Kaoutar Aboukhalid, Rachid Sabbahi, Sabir Ouahhoud, Sanae Baddaoui, Abdessadek Essadek, Abdesselam Maatougui, Ennouamane Saalaoui, Mounsef Neffa

**Affiliations:** 1 Laboratory of Bioresources, Biotechnology, Ethnopharmacology and Health, Faculty of Sciences, Mohammed First University, BV Mohammed VI BP 717, Oujda 60000, Morocco; abderrahman.makaoui@etu.uae.ac.ma (A.M.); sanaebaddaoui@gmail.com (S.B.); a.essadek@ump.ac.ma (A.E.); e.saalaoui@ump.ac.ma (E.S.); 2 Physical Chemistry of Natural Substances and Process Team, Laboratory of Applied Chemistry and Environment (LCAE-CPSUNAP), Department of Chemistry, Faculty of Sciences, Mohammed First University, BV Mohammed VI BP 717, Oujda 60000, Morocco; talhaouiabdelmonaem@gmail.com; 3 National Institute of Agronomic Research, CRRA Oujda, 10 Bd Mohamed VI BP 428, Oujda 6000, Morocco; k.aboukhalid@yahoo.com (K.A.); abdesselam.maatougui@inra.ma (A.M.); 4 Research Team in Science and Technology, Higher School of Technology of Laayoune, Ibn Zohr University, Laayoune P.O. Box 3007, Morocco; 5 Laboratory of Health Sciences, Artificial Intelligence, and Applied Nanotechnology, Faculty of Medicine and Pharmacy, University Sultan Moulay Slimane, Beni Mellal 23000, Morocco; s.ouahhoud@ump.ac.ma

**Keywords:** antioxidant activity, chemical composition, essential oils, Lamiaceae family, genotoxicity, DPPH, β-carotene bleaching, FRAP

## Abstract

This study investigated the chemical composition, antioxidant activity, and genotoxic potential of essential oils (EOs) obtained by hydrodistillation from aerial parts of four wild-growing Lamiaceae species in eastern Morocco: Spanish ziziphora (*Ziziphora hispanica* L.), felty germander (*Teucrium polium* L.), French lavender (*Lavandula dentata* L.), and topped lavender (*Lavandula stoechas* L.). Gas chromatography–mass spectrometry (GC-MS) analysis revealed eucalyptol (40.08%), thujopsene (11.25%), β-myrcene (15.82%), and fenchone (30.69%) as the major constituents in *Z. hispanica*, *T. polium*, *L. dentata*, and *L. stoechas*, respectively. Antioxidant capacity was evaluated using three complementary assays: 2,2-diphenyl-1-picrylhydrazyl radical scavenging, ferric reducing antioxidant power, and β-carotene bleaching. *L. stoechas* and *L. dentata* exhibited the strongest antioxidant activities, with IC_50_ values ranging from 0.284 to 1.71 mg/mL across assays. Genotoxicity was assessed in rat leukocytes using the alkaline Comet assay at EO concentrations of 2.5, 5, and 10 µg/mL. All tested EOs induced statistically significant DNA damage compared to the negative control, though the extent varied by species and concentration; notably, *L. stoechas* at 2.5 µg/mL showed the lowest genotoxic impact. These findings highlight the dual potential of these EOs as natural antioxidants while underscoring the need for dose-dependent safety evaluation prior to therapeutic or industrial application. Given that DNA damage was detectable even at 2.5 µg/mL, a conservative practical recommendation is to keep EO levels below 2.5 µg/mL-equivalent in preliminary applications, pending further in vivo toxicology to establish NOAEL-based exposure limits.

## 1. Introduction

The Mediterranean region is home to a diverse and fragrant flora, including several species of the Lamiaceae family. This family, also known as the mint family, comprises over 7000 species in about 240 genera, many of which have economic and medicinal value [1]. Notable members of this family include Spanish ziziphora (*Ziziphora hispanica* L.), felty germander (*Teucrium polium* L.), French lavender (*Lavandula dentata* L.), and topped lavender (*Lavandula stoechas* L.). These plants are widely utilized in cosmetics, traditional medicine, aromatherapy, and perfumes because of their rich content of phenolic constituents and essential oils (EOs). These natural compounds offer a range of health benefits, including protection against mutations, reduction in inflammation, defense against oxidative damage, cancer prevention, and support for brain health [2,3,4,5].

*Z. hispanica* is a perennial herb with minty and aromatic leaves that is used as a culinary herb, a tea ingredient, and a remedy for various ailments. Similarly, *T. polium* is a tiny shrub with purple flowers and felty-textured leaves that is used as an antidiabetic, anti-inflammatory, antispasmodic, and antiseptic agent, as well as a wound healer and a liver tonic. On the other hand, *L. dentata* is an evergreen shrub with serrated leaves and purple flowers that is cultivated for its ornamental and aromatic value. It is used as a relaxant, an antidepressant, an antiseptic, and an insect repellent [6,7]. Another native shrub is *L. stoechas*, which has linear leaves and purple flowers with a tuft of bracts on top. It is traditionally used for ornamental and aromatic purposes, as well as for relieving pain, preventing infection, reducing swelling, and easing gas. However, caution is advised before using these plants for medicinal or culinary purposes, as they may have adverse effects or interactions if not used properly. Therefore, professional guidance is recommended before employing these plants [8].

The EO of the Lamiaceae plants are volatile liquids that can evaporate easily and can be obtained from different parts of the plants, such as leaves, roots, flowers, seeds, and fruits, using different techniques, such as steam distillation, CO_2_ supercritical extraction, and hydrodistillation [1]. These oils have a long history of use in traditional medicine, especially in the Mediterranean region, where they are valued for their therapeutic properties. The EOs of these plants have been extensively studied for their phytochemical composition, which reveals the presence of various constituents, like carvacrol, eucalyptol, pulegone, fenchone, and β-pinene. For instance, the EOs of *L. dentata* and *L. stoechas* contain approximately 52 phytochemical compounds [9]. Carvacrol, eucalyptol, and fenchone are the major phytochemical compounds in these species, respectively [8,10]. Alternatively, *Z. hispanica* EO may contain bioactive compounds that can prevent oxidative stress and bacterial infections with their antioxidant and antibacterial properties. The EO from this plant has shown an antibacterial activity towards *Listeria monocytogenes*, *Enterococcus faecalis* and *Pseudomonas aeruginosa*, and a bacteriostatic activity towards *Staphylococcus aureus*. Moreover, they had a strong ability to scavenge DPPH (2,2-diphenyl-1-picrylhydrazyl) radical (IC_50_ = 1.3 mg/mL) [11,12,13].

In Eastern Morocco (Tafoughalt/Berkane area), *Z. hispanica*, *T. polium*, *L. dentata*, and *L. stoechas* are among the commonly used aromatic Lamiaceae species in traditional practices and perfumery [14,15,16]. However, for these local wild chemotypes, comparative datasets that jointly address (i) detailed Gas chromatography–mass spectrometry (GC-MS) composition, (ii) antioxidant performance across complementary mechanisms, and (iii) genotoxic safety in a dose-aware manner remain scarce. Many available reports focus on chemical profiling and/or a single biological endpoint, and dose–response interpretation of DNA-damage signals is rarely discussed for EOs.

Therefore, the objectives of the present study were to: (i) characterize the chemical composition of EOs from four Lamiaceae species harvested in Eastern Morocco using GC-MS; (ii) evaluate antioxidant capacity using three complementary assays (DPPH, FRAP, and β-carotene bleaching); and (iii) assess genotoxicity using the alkaline Comet assay in rat leukocytes at 2.5, 5, and 10 µg/mL. The novelty of this work lies in providing a harmonized, comparative chemistry-to-biology evaluation that integrates antioxidant potential with dose-dependent genotoxicity screening for Eastern Morocco wild populations, thereby supporting more evidence-based consideration of both benefits and safety constraints prior to application.

## 2. Results and Discussion

### 2.1. Chemical Profile of EO

**Figure 1:**
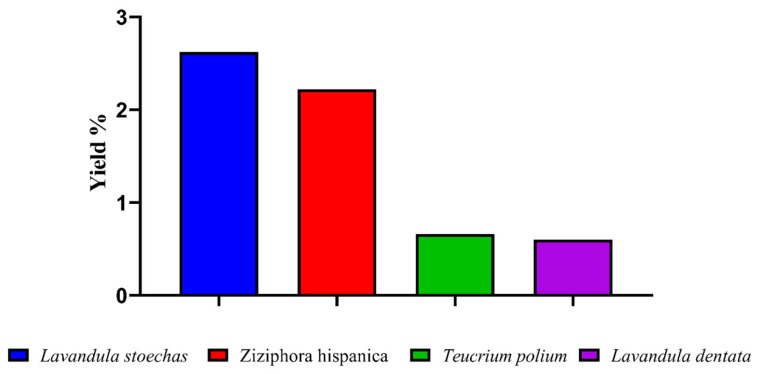
Essential oil yield of *Lavandula stoechas*, *Ziziphora hispanica*, *Teucrium polium*, and *Lavandula dentata*.

The EO yield of the four studied plant species ranged from 0.60 to 2.62%. *L. stoechas* exhibited the highest yield, followed by *Z. hispanica*, whereas *T. polium* and *L. dentata* showed the lowest yields (Figure 1). Comparable yields have been reported for these species collected from distinct geographic areas. For instance, the EO yields of *Z. hispanica* and *L. dentata* harvested in Tlemcen (Algeria) and Imouzzer Kandar (Morocco) were 0.53 and 3.46%, respectively [17,18]. Likewise, the EO yields of *L. stoechas* and *T. polium* collected in Kairouan (Tunisia) and M’Sila (Algeria) were 0.77 and 0.53%, respectively [13,19]. These inter-study differences likely reflect the combined influence of harvesting region and altitude, which shape local microclimatic conditions (e.g., temperature regime, rainfall/humidity, solar radiation/UV exposure, and wind), as well as edaphic factors (soil type, mineral availability, and water status). In addition, EO accumulation is strongly affected by harvesting time and phenological stage (pre-flowering, flowering, or post-flowering) and may also vary with year-to-year climate variability and post-harvest handling prior to distillation. Therefore, the variability observed across regions and altitudes supports the conclusion that environmental conditions and harvest timing are key drivers of EO yield in these Lamiaceae species.

**Figure 2:**
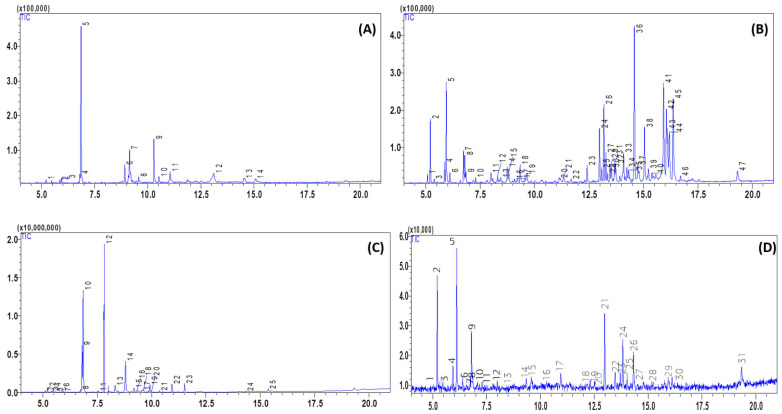
GC-MS chromatogram of *Ziziphora hispanica* (**A**), *Teucrium polium* (**B**), *Lavandula stoechas* (**C**), and *Lavandula dentata* (**D**) essential oil extracts; blue trace corresponds to the TIC (total ion chromatogram) signal in the GC–MS chromatograms.

The GC-MS determined the chemical composition of EO (Table 1). To provide a clearer overview of the oil profiles, a summary of the major constituents is provided in Table S1. *Z. hispanica* EO was dominated by eucalyptol (monoterpene), which accounted for 40.08% of the total composition (Figure 2A). Other notable components were menthol (a monoterpene) at 10.77%, d-p-Menth-4(8)-en-3-one at 12.74%, and (R)-(+)-3-methyladipic acid at 10.37%. This composition is different from the one reported by Bekhechi et al. [18] for the Algerian *Z. hispanica* EO, in which pulegone was the primary component (79.5%), along with limonene (1.7%), carvacrol (1.6%) and thymol (0.9%).

The EO of *T. polium* (Figure 2B) contained 47 compounds. The main component was thujopsene (sesquiterpene), which represented 11.25% of the total composition. Other significant components were tau-cadinol (sesquiterpene) at 8.84%, α-bisabolol (sesquiterpene) at 8.01%, and β-pinene (bicyclic monoterpene) at 5.43%. Chabane et al. [12] also found that the *T. polium* EO obtained in M’sila (Algeria) had a high monoterpene content, especially β-pinene (32.8%), germacrene D (16.6%), α-pinene (9.7%) and myrcene (7.8%). Another study reported similar percentages of α-pinene (6.97%), β-pinene (12.97%) and myrcene (2.19%) in the EO of *T. polium* leaves collected in Behbahan city [20].

The GC-MS analysis of *L. stoechas* EO revealed 25 compounds (Figure 2C). The main component was fenchone (a bicyclic monoterpene ketone), which accounted for 30.69% of the total oil. Other important compounds were eucalyptol (25.04%) and camphor (bicyclic monoterpene ketone) (11.77%). Bouzouita et al. [19] analyzed the EO of *L. stoechas* leaves harvested in Kairouan, Tunisia and found fenchone (68.2%) and camphor (11.2%) as the major components. Similarly, Zohra and Fawzia [21] studied the EO of *L. stoechas* leaves collected in Tlemcen, Algeria, and identified fenchone (27.6%), eucalyptol (18.9%) and camphor (18.1%) as the main constituents.

The EO of *L. dentata* consisted of 30 different compounds (Figure 2D). The predominant compound was β-myrcene, which represented 15.82% of the EO, followed by caryophyllene (12.10%), α-pinene (12.09%), germacrene D (8.96%), and (+)-delta-Cadinene (6.10%). El Abdali et al. [22] studied the EO of *L. dentata* leaves collected in Imouzzer Kandar, Morocco and found borneol (8.28%), camphor (15.62%), and linalool (45.06%) as the main components. These variations in EO composition can be ascribed to the combined effects of geographical origin, climate, altitude, and genetic background. Geographical location is often associated with differences in edaphic conditions (soil texture, pH, nutrient availability, and water status) that influence plant physiology and the biosynthesis of volatile secondary metabolites. Climatic parameters such as temperature, rainfall/humidity, solar radiation, and seasonal variability can modulate terpene metabolism, often shifting the balance between hydrocarbon and oxygenated mono- and sesquiterpenes. Altitude may further reshape EO profiles through changes in UV exposure, day–night thermal amplitude, and atmospheric conditions, which can promote the accumulation of specific protective terpenoids and lead to distinct compositional patterns across elevation gradients. In addition to environmental effects, genetic variability and chemotype differentiation within the same species are key drivers of EO diversity, as differences in terpene synthase expression and pathway fluxes can result in different dominant constituents even under comparable growing conditions. Finally, EO composition also depends on harvesting time and phenological stage (e.g., pre-flowering vs. flowering), which can alter the relative abundance of major compounds and contribute to inter-study variability [20,21,22,23,24,25,26,27,28,29].

**Table 1:** Chemical composition (relative area, %) of *Ziziphora hispanica*, *Teucrium polium*, *Lavandula stoechas*, and *Lavandula dentata* essential oils.

Compound	RI_EXP_	RI_LIT_	RT			Area (%)			Reference
				*Ziziphora hispanica*	*Teucrium polium*	*Lavandula stoechas*	*Lavandula dentata*	Method of Identification	
Santolina triene	906	906	5.077	-	-	0.98	-	RI, MS	[30]
∝-Thujene	925	930	5.078	-	0.50	-	0.01	RI, MS	[31]
Tricyclene	930	921	5.205	-	-	0.91	-	RI, MS	[32]
∝-Pinene	934	939	5.217	1.20	2.94	0.43	12.09	RI, MS	[31]
Camphene	951	954	5.388	-	-	0.34	1.17	RI, MS	[31]
Sabinene	972	969	5.862	0.93	1.18	-	-	RI, MS	[31]
β-Pinene	970	979	5.936	1.26	5.43	1.36	3.71	RI, MS	[31]
β-Myrcene	979	988	6.110	-	1.70	-	15.82	RI, MS	[30]
∝-Phellandrene	1008	1000	6.398	-	-	-	1.44	RI, MS	[33]
∝-Terpinene	1016	1017	6.591	-	-	-	0.37	RI, MS	[31]
*p*-Cymene	1025	1027	6.735	-	1.76	-	1.58	RI, MS	[32]
d-Limonene	1031	1030	6.799	2.27	1.58	-	7.27	RI, MS	[34]
Eucalyptol	1032	1016	6.855	40.08	1.66	25.04	-	RI, MS	[33]
Cis-β-Ocimene	1038	1039	7.077	-	-	-	1.17	RI, MS	[32]
Trans-Verbenol	1145	1148	7.164	-	0.61	4.06	-	RI, MS	[34]
γ-Terpinen	1059	1059	7.291	-	0.40	-	1.38	RI, MS	[31]
Linalool, oxide	1080	1081	7.553	-	-	1.30	-	RI, MS	[35]
Fenchone	1092	1094	7.832	-	-	30.69	-	RI, MS	[32]
Linalool <tetrahydro->	1096	1098	7.993	-	0.83	-	-	RI, MS	[35]
Isoamyl Isovalerate	1102	1092	7.999	-	-	-	0.07	RI, MS	[36]
β-Thujone	1115	1114	8.295	-	0.58	-	-	RI, MS	[37]
*Trans*-*p*-Mentha-2,8-dienol	1120	1124	8.320	-	1.18	-	-	RI, MS	[38]
Fenchol	1121	1123	8.330	-	-	5.01	-	RI, MS	[32]
Camphenol	1125	1110	8.449	-	0.58	-	-	RI, MS	[39]
*Trans*-Pinocarveol	1130	1139	8.741	-	1.15	1.20	-	RI, MS	[37]
δ-2-Carene	1139	1040	7.794	-	-	-	1.20	RI, MS	[40]
Camphor	1143	1146	8.802	-	-	11.77	-	RI, MS	[31]
Verbenol	1143	1146	8.812	-	0.93	-	-	RI, MS	[32]
*p*-Menthan-3-one	1167	1158	8.915	5.88	-	-	-	RI, MS	[41]
Pinocarvone	1160	1164	9.080	-	1.28	-	-	RI, MS	[31]
Menthol	1161	1167	9.136	10.77	-	-	-	RI, MS	[42]
∝-Terpineol	1172	1189	9.568	-	-	0.83	2.11	RI, MS	[31]
Myrtenol	1175	1195	9.626	-	-	1.26	-	RI, MS	[31]
4-Terpineol	1182	1177	9.635	-	1.44	1.11	-	RI, MS	[31]
4-Carvomenthenol	1195	1192	9.642	-	-	-	2.03	RI, MS	[43]
2-Pinen-10-ol	1199	1196	9.640	-	1.22	1.4	-	RI, MS	[44]
*Trans*-Carveol	1215	1217	9.920	-	-	0.62	-	RI, MS	[37]
Fenchyl acetate	1228	1227	10.04	-	-	2.09	-	RI, MS	[32]
d-*p*-Menth-4(8)-en-3-one	1233	1233	10.287	12.74	-	-	-	RI, MS	[45]
Carvone	1242	1247	10.364	-	-	0.71	-	RI, MS	[42]
Isopentyl hexanoate	1250	1251	10.466	-	-	-	0.20	RI, MS	[46]
*Cis*-Piperitone Époxide	1252	1250	10.325	1.69	-	-	-	RI, MS	[47]
Bicyclo [3.2.0] heptan-2-one, 5-formylmethyl-6-hydroxy-3,3-dimethyl-6-vinyl-	1254	1254	11.048	4.51	-	-	-	RI, MS	[48]
Linalyl acetate	1255	1254	11.082	-	-	0.29	-	RI, MS	[49]
*Cis*-Chrysanthenyl acetate	1260	1262	11.107	-	-	0.87	-	RI, MS	[50]
Bornyl acetate	1282	1285	11.110	-	-	2.38	3.84	RI, MS	[32]
1-Decanol	1286	1276	11.112	3.47	-	-	-	RI, MS	[51]
*p*-Cymen-7-ol	1289	1305	11.128	-	1.38	-	-	RI, MS	[52]
Thymol	1290	1310	11.333	-	0.91	-	-	RI, MS	[52]
(R)-(+)-3-Methyladipic acid	1294	1315	11.407	10.37	-	-	-	RI, MS	[53]
*p*-Menth-1-en-8-ol	1296	1296	11.465	2.09	-	-	-	RI, MS	[54]
Myrtenyl acetate	1312	1332	11.518	-	-	3.11	-	RI, MS	[55]
β-Cubebene	1367	1383	11.878	-	-	-	0.48	RI, MS	[56]
∝-Copaene	1372	1376	12.311	-	-	-	1.92	RI, MS	[35]
Isoledene	1407	1407	12.321	-	-	0.41	-	RI, MS	[57]
1,7-di-épi-α-Cédrène	1393	1393	12.891	-	2.34	-	-	RI, MS	[58]
β-Caryophyllen	1418	1420	12.980	-	3.51	-	-	RI, MS	[34]
Caryophyllene	1420	1413	13.014	-	-	-	12.10	RI, MS	[59]
∝-Bergamotene	1426	1436	13.078	-	1.07	-	-	RI, MS	[60]
β-Cedrene	1429	1418	13.181	-	4.89	-	-	RI, MS	[61]
T-Muurolol	1444	1443	13.240	-	-	-	2.75	RI, MS	[62]
β-Farnesene	1458	1462	13.275	-	1.77	-	-	RI, MS	[63]
1,5,9,9-Tetramethyl-1,4,7-cycloundecatriene	1468	1438	13.461		1.06	-	3.28	RI, MS	[35]
γ-Muurolene	1478	1477	13.713	-	1.18	-	3.41	RI, MS	[35]
∝-Curcumene	1479	1485	13.720	-	1.56	-	-	RI, MS	[35]
Germacrene D	1480	1481	13.849	-	-	-	8.96	RI, MS	[34]
∝-Zingiberene	1487	1487	12.410	-	1.21	-	-	RI, MS	[64]
∝-Muurolene	1495	1507	14.031	-	-	-	3.41	RI, MS	[35]
Thujopsene	1497	1497	14.052	-	11.25	-	-	RI, MS	[65]
β-Himachalene	1500	1510	14.068	-	1.17	-	-	RI, MS	[66]
β-Sesquiphellandrene	1521	1523	14.308	-	1.70	-	-	RI, MS	[67]
δ-Cadinene	1515	1514	14.315	-	1.92	-	-	RI, MS	[34]
δ-Amorphene	1523	1525	14.339	-	-	-	6.10	RI, MS	[68]
Globulol	1580	1585	15.366	-	-	1.00	-	RI, MS	[35]
Caryophyllene oxide	1585	1585	15.231	-	-	-	0.23	RI, MS	[35]
T-Cadinol	1640	1644	15.925	-	8.84	-	-	RI, MS	[34]
Bisabolol oxide	1646	1651	16.054	-	5.71	-	-	RI, MS	[69]
∝-Cadinol	1651	1650	16.102	-	3.57	-	-	RI, MS	[32]
β-Bisabolol	1662	1662	16.194	-	5.04	-	-	RI, MS	[70]
∝-Bisabolol	1680	1682	16.362	-	8.01	-	-	RI, MS	[35]
8-Cedren-13-ol	1688	1688	16.590	-	1.00		-	RI, MS	[71]
perillyl Alcohol	1780	1779	16.677	-	0.46	-	-	RI, MS	[72]
Eicosanoic acid	1988	1986	17.321	-	1.26	-	-	RI, MS	[71]
Bicyclo [5.3.0]decane, 2-methylene-5-(1-methylvinyl)-8-methyl-	2000	2005	18.058	-	-	-	0.94	RI, MS	[72]
% identification				97.26%	96.54%	99.68%	99.03%		

RI_EXP_ = experimental retention index; RI_LIT_ = literature retention index from the Adams, Wiley and NIST libraries, supported by bibliographic references; and RT= retention time.

### 2.2. Antioxidant Potential of EO

#### 2.2.1. DPPH Free Radical Scavenging Assay

**Figure 3:**
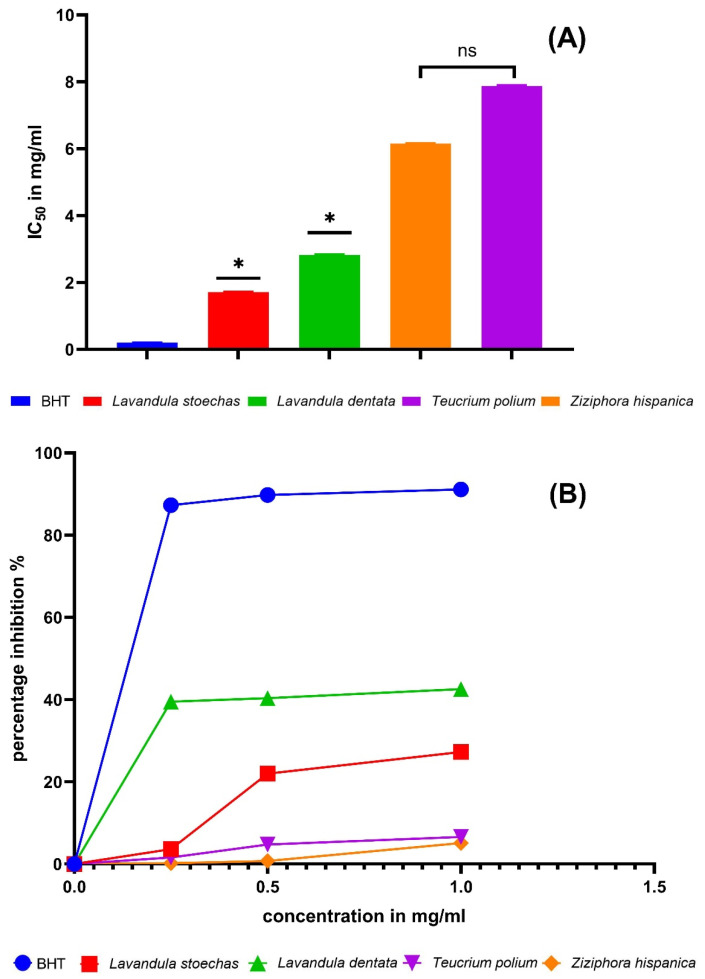
Essential oil antioxidant activity measurement by the DPPH method. Each value is expressed as mean ± standard deviation (*: *p* ≤ 0.05, ns: *p* > 0.05, (**A**) IC_50_ in mg/mL, and (**B**) percentage of inhibition).

Among the four EOs, *L. stoechas* and *L. dentata* showed the lowest IC_50_ values (highest DPPH scavenging capacity), whereas *Z. hispanica* and *T. polium* displayed higher IC_50_ values (lower activity) (Figure 3). These inter-species differences can be largely attributed to qualitative and quantitative variations in EO composition, since the DPPH assay primarily reflects the ability of constituents to donate hydrogen atoms or electrons. In EOs, stronger DPPH scavenging is often associated with the presence of highly reactive oxygenated and/or phenolic constituents and with synergistic interactions among minor compounds. In our samples, the comparatively higher activity of *L. stoechas* may relate to its richness in oxygenated monoterpenes/ketones (e.g., fenchone and camphor) together with other oxygenated constituents, while *L. dentata* contains a distinct mixture of mono- and sesquiterpenes (e.g., β-myrcene, caryophyllene, α-pinene, germacrene D) that may contribute additively to radical scavenging. In contrast, the higher IC_50_ values observed for *Z. hispanica* and *T. polium* are consistent with EOs dominated by constituents that generally exhibit more moderate DPPH reactivity when present without strongly donating phenolic compounds, and may also reflect differences in the relative abundance of minor antioxidants. Importantly, published IC_50_ values for these species vary widely (e.g., *L. stoechas* [20], *T. polium* [12], and *L. dentata* [22]), which likely arises from chemotype/genetic variability, geographical origin, and environmental conditions (altitude, temperature, rainfall, and harvest stage), as well as assay-dependent factors such as solvent/emulsification and experimental settings. Therefore, the observed ranking among species in our study is best interpreted as a consequence of both compositional differences and inter-study methodological variability, underscoring the need to compare antioxidant metrics in light of EO chemotypes and test conditions.

#### 2.2.2. β-Carotene Bleaching Test

**Figure 4:**
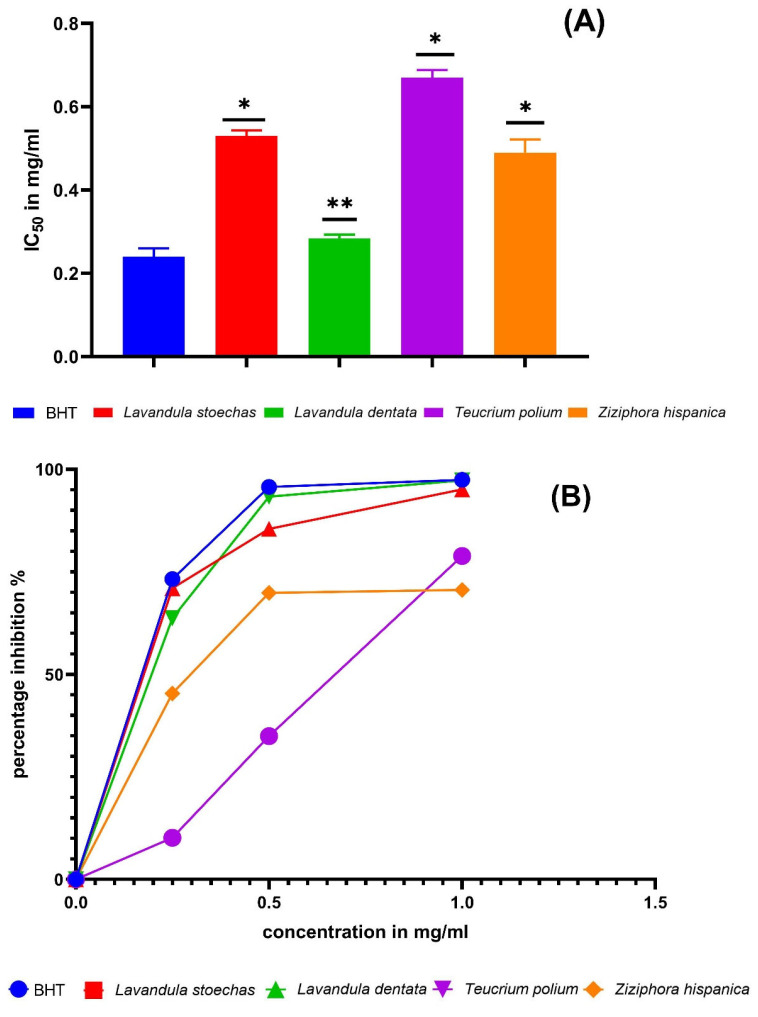
Essential oil antioxidant activity measurement by the β-carotene bleaching assay. Each value is expressed as mean ± standard deviation (*: *p* ≤ 0.05, **: *p* ≤ 0.01, (**A**) IC_50_ in mg/mL, and (**B**) percentage of inhibition).

The IC_50_ values obtained from the β-carotene bleaching assay for each EO and the BHT (control) were *L. stoechas* (0.53 ± 0.01 mg/mL), *L. dentata* (0.284 ± 0.009 mg/mL), *T. polium* (0.67 ± 0.018 mg/mL), *Z. hispanica* (0.49 ± 0.013 mg/mL), and BHT (0.24 ± 0.02 mg/mL) (Figure 4). *Z. hispanica*, *T. polium*, and *L. stoechas* EO had comparable antioxidant activity to BHT (*p* < 0.05). However, the EO of *L. dentata* showed a significant increase in antioxidant activity compared to BHT (*p* < 0.01), indicating that it was as effective as BHT. These findings concur with those of earlier studies that reported the EO antioxidant activity of these plants collected from different regions. For example, the EO isolated from *L. stoechas* leaves collected in northern Morocco showed 58.16 ± 1.4% inhibition at 10 μL/mL [10]. In addition, a study conducted by Bakari et al. [72] showed that EO of *T. polium* collected in the region of Kef, in southwest Tunisia, had an IC_50_ value of 0.15 mg/mL.

#### 2.2.3. FRAP Test

**Figure 5:**
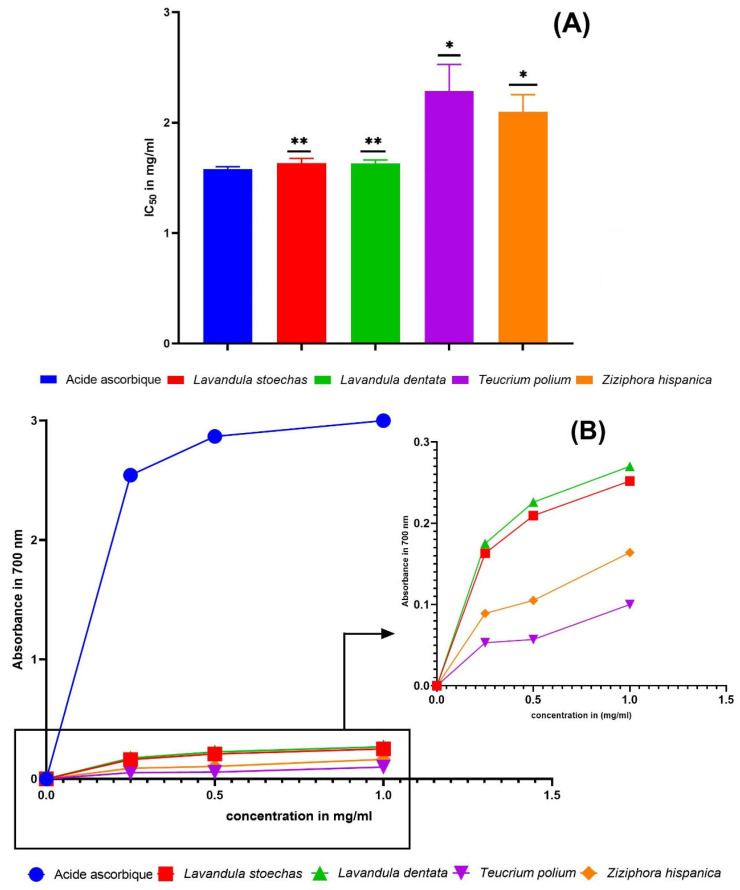
Essential oil antioxidant activity measurement by the FRAP method. Each value is expressed as mean ± standard deviation (**: *p* ≤ 0.01, *: *p* ≤ 0.05, (**A**) IC_50_ in mg/mL, and (**B**) percentage of inhibition).

The IC_50_ values obtained from the FRAP assay for the EO and the ascorbic acid control were *L. stoechas* (1.632 ± 0.045 mg/mL), *L. dentata* (1.63 ± 0.0322 mg/mL), *T. polium* (2.287 ± 0.024 mg/mL) and *Z. hispanica* (2.099 ± 0.01556 mg/mL), and ascorbic acid (1.578 ± 0.023 mg/mL) (Figure 5). The results showed that the EO of *Z. hispanica* and *T. polium* had similar antioxidant activity to ascorbic acid (*p* < 0.05). On the other hand, *L. dentata* and *L. stoechas* EO had significant antioxidant activity, belonging to the same group but with a *p* < 0.01, indicating that they were as effective as ascorbic acid. These findings align with earlier studies that reported the EO antioxidant activity of these plants collected from different sources. For instance, Touaibia et al. [73] focused on the EO extracted from *T. polium* aerial parts collected in the Djelfa province of Algeria. Using the FRAP test, they found an IC_50_ of 0.98 ± 0.70 mg/mL. Similarly, Ghanimi et al. [9] examined the EO of *L. stoechas* and *L. dentata* collected in Al-Haouz region of Morocco. They reported that IC_50_ values were 6.88 ± 0.8 and 9.23 ± 1.4 mg/mL, respectively.

The observed differences in IC_50_ values among the tested EO can be attributed to their phenolic compound content. Phenols are known for their potent antioxidant properties, and therefore, EO with higher phenolic compound content tends to exhibit enhanced antioxidant activity. However, several factors can influence the IC_50_ values, including the concentration of DPPH, FRAP and β-carotene solutions and incubation time. Additionally, variability in results can also be affected by the diverse chemical composition of EO and intrinsic and extrinsic factors, such as cultivar, region, and extraction method. Hence, these findings suggest the need for further research on the antioxidant potential of these EO and their possible applications in various fields [74].

#### 2.2.4. Linking Chemical Composition to Biological Effects and Structure-Activity Considerations

The three antioxidant assays used in this study probe complementary mechanisms (radical scavenging in DPPH, reducing power in FRAP, and inhibition of lipid peroxidation in the β-carotene/linoleic acid model). Accordingly, differences among EOs are best interpreted by considering dominant chemical classes and mixture-level interactions rather than attributing activity to a single compound.

In our GC-MS profiles, *Z. hispanica* was dominated by the oxygenated monoterpene eucalyptol, *L. stoechas* by the bicyclic monoterpene ketone fenchone, *L. dentata* by the monoterpene hydrocarbon β-myrcene, and *T. polium* by the sesquiterpene thujopsene. Oxygenated monoterpenes may contribute to redox- and membrane-related effects that are particularly relevant in lipid-peroxidation settings, whereas sesquiterpene-rich profiles can modulate cellular stress responses in a concentration-dependent manner. These compositional differences provide a plausible basis for the observed variability across DPPH/FRAP/β-carotene assays.

From a structure-activity perspective, strong DPPH-type radical scavenging is often associated with phenolic OH-bearing monoterpenes (e.g., thymol/carvacrol) due to efficient hydrogen donation and resonance stabilization; however, such phenolic constituents were minor in the oils analyzed here. Therefore, the antioxidant activity observed is likely driven by combined contributions of non-phenolic terpenoids and mixture interactions (additive/synergistic or antagonistic effects). Regarding genotoxicity, some terpenoids may, at sufficiently high concentrations, promote oxidative imbalance or perturb membranes, indirectly affecting DNA integrity. We therefore present composition–effect links as association-level interpretations within the tested exposure range, and note that fractionation and/or authentic standards will be required to confirm which constituents drive the observed Comet assay responses.

### 2.3. Genotoxic Effect of EO

**Figure 6:**
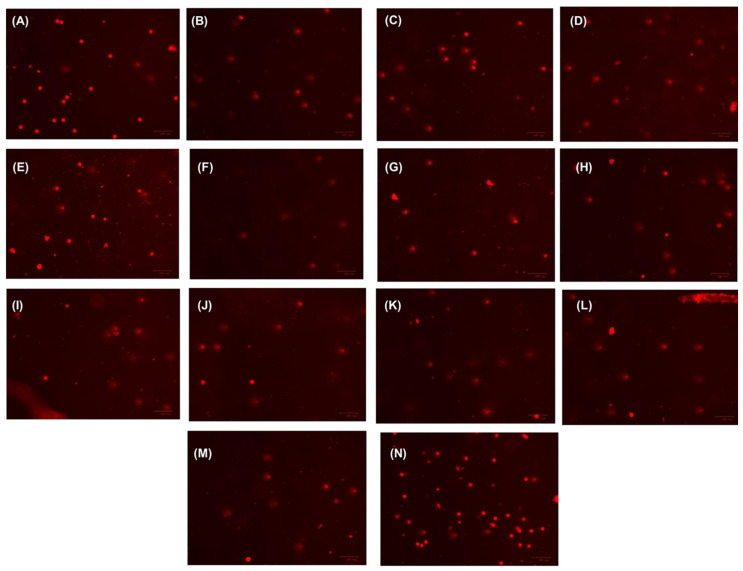
Leukocyte DNA damage detection by the Comet assay using fluorescence microscopy (**A**) negative control; (**B**–**D**) *L. stoechas* at 2.5, 5, and 10 µg/mL; (**E**–**G**) *L. dentata* at 2.5, 5, and 10 µg/mL; (**H**–**J**) *Z. hispanica* at 2.5, 5, and 10 µg/mL; (**K**–**M**) *T. polium* at 2.5, 5, and 10 µg/mL; and (**N**) H_2_O_2_ group at 250 µM.

**Figure 7:**
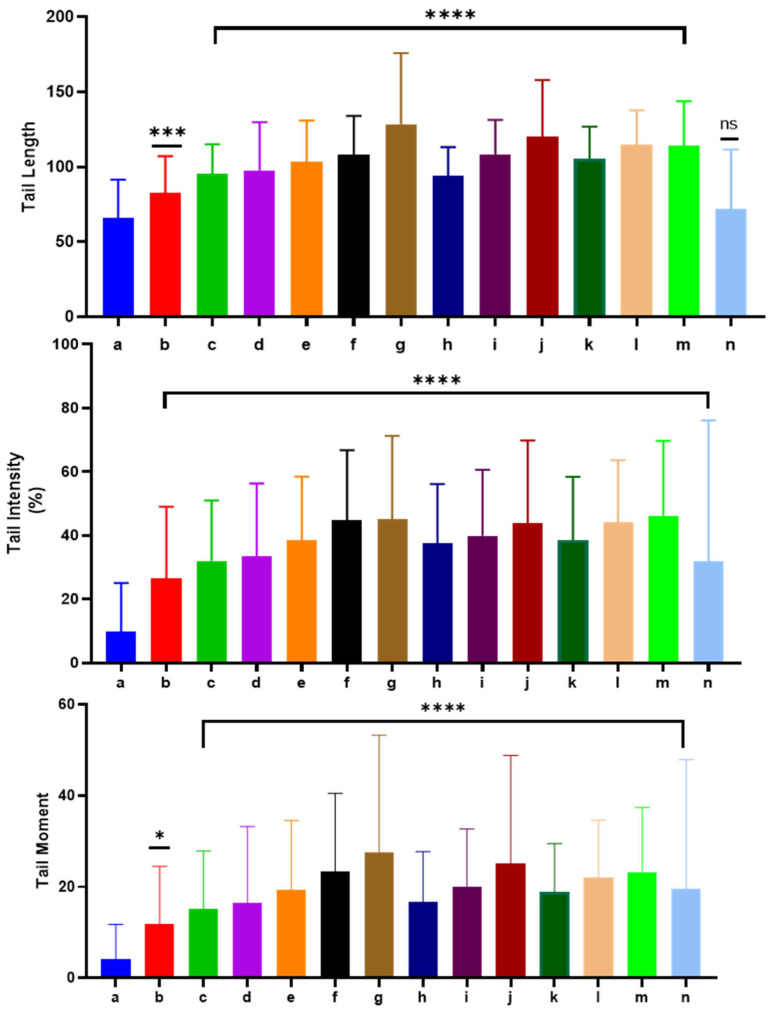
Evaluation of genotoxic effects of essential oils of studied species using Comet assay (a: negative control; b, c and d: *Lavandula stoechas* at 2.5, 5, and 10 µg/mL; e, f and g: *Lavandula dentata* at 2.5, 5, and 10 µg/mL; h, i and j: *Ziziphora hispanica* at 2.5, 5, and 10 µg/mL; k, l and m: *Teucrium polium* at 2.5, 5, and 10 µg/mL; n: H_2_O_2_ group at 250 µM; ****: *p* ≤ 0.0001; ***: *p* ≤ 0.001; *: *p* ≤ 0.05; and ns: *p* > 0.05).

The Comet assay, also known as single-cell gel electrophoresis, is a highly adaptable method for evaluating DNA strand breaks in individual cells. This technique can be applied to both cultured cells and cells taken from organisms that have been exposed to various treatments. It allows for precise and sensitive detection of genetic damage, offering a detailed view of DNA alterations caused by external agents like chemicals or radiation. Due to its versatility, the Comet assay is extensively utilized in genotoxicity research, biomedical studies, and assessing the environmental impact of various substances [75,76,77]. In our study, we investigated the genotoxicity of EOs extracted from four species within the Lamiaceae family using the Comet assay. Leukocyte DNA damage was visualized through fluorescence microscopy (Figure 6). The genotoxicity of the EOs was evaluated at 2.5, 5, and 10 µg/mL, a concentration range selected to allow for a dose–response assessment while remaining below the threshold of excessive cytotoxicity, which can confound Comet assay results. As shown in Figure 7 and Table S2, a clear dose-dependent increase in DNA migration was observed across all species. Regarding tail moment, even the lowest concentration of *L. stoechas* (2.5 µg/mL) showed a significant increase compared to the negative control (*p* = 0.0132), while all higher concentrations (5 and 10 µg/mL) for all four species reached a highly significant deviation (*p* < 0.0001). Similar trends were observed for tail intensity and tail length, where all tested concentrations triggered significant DNA damage compared to the negative control (*p* < 0.0001). Notably, while the effects were significant compared to the baseline, the levels of DNA damage remained significantly lower than those induced by the positive control (H2O2, 250 µM), suggesting that while these EOs exhibit genotoxic potential at the tested doses, the intensity of the effect is concentration-governed.

Medicinal plants can exhibit genotoxic effects if they contain toxic compounds to DNA, such as certain phenolic or monoterpene compounds. The review by Bardoloi and Soren [78] focused on medicinal plants exerting a genotoxicity effect. They revealed that the Lamiaceae plant family is classified as having a higher frequency of genotoxicity. Similarly, Nikolić et al. [79] conducted a study to evaluate the biological activity of the EO of five species in the Lamiaceae family. Their findings indicated that one of the oils tested exhibited strong cytotoxic activity against the cell lines tested, with IC50 values ranging from 40.13 to 65.51 μg/mL. Slight selectivity was observed, favoring HeLa cells over the normal cell line (MRC-5).

Furthermore, Damasceno et al. [80] demonstrated that all the compounds tested and EO of the Lamiaceae family showed low cytotoxic activity against the cell lines tested. Indeed, the genotoxicity of some EO is confirmed by other research, such as the study by Péres et al. [81], which demonstrates the potent genotoxic, cytotoxic, and mutagenic effects of *Piper gaudichaudianum* Kunth EO in V79 cells, which may be attributed to its oxidative damage. The same study showed that DNA damage starts at concentrations of up to 2 μg/mL. However, not all EO are inherently genotoxic, and the degree of genotoxicity can vary from one oil to another. It is important to note that the concentrations used in our study may be relatively high and may not reflect those typically used in common EO applications. It is, therefore, essential to follow recommended doses and precautions to avoid adverse reactions. Additionally, further studies are needed to better understand each EO’s effects on living cells. From a safety perspective, the present Comet assay results indicate that genotoxic effects can be detected even at the lowest tested concentration (2.5 µg/mL), highlighting the importance of a precautionary approach when considering these EOs in preliminary applications. Although in vitro concentrations cannot be directly extrapolated to human exposure levels, this finding supports the use of low dilutions and strict risk management in consumer products. In practice, for dermal consumer formulations, EOs are typically used at low concentrations in finished products, for example, ~0.1–1.0% in facial/sensitive leave-on products and ~1–2% in general leave-on body products, with up to ~3% in rinse-off products depending on the specific oil/constituent profile and product category. Importantly, maximum use levels should comply with established safety frameworks where applicable (e.g., product-category restrictions and constituent-based limits), and additional in vivo toxicological studies are required to derive NOAEL-based exposure limits and define robust recommended dose ranges for different routes of use.

## 3. Materials and Methods

### 3.1. Materials

**Figure 8:**
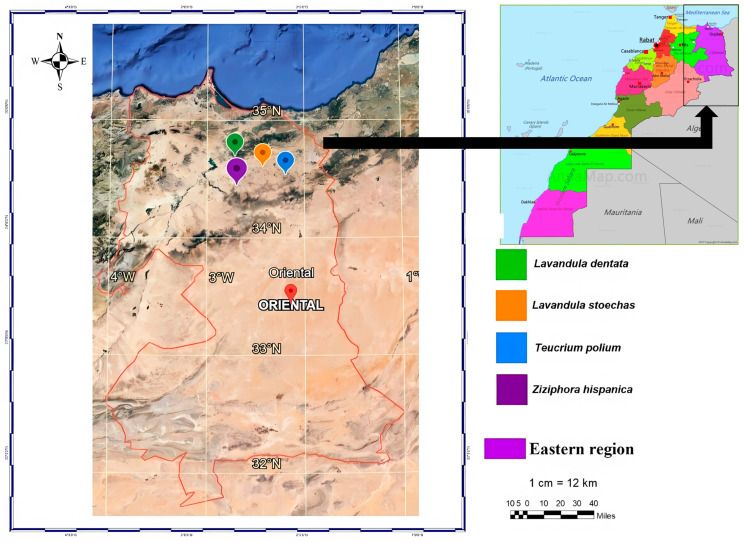
Map showing locations of the collected plant species.

Aerial parts of *Z. hispanica*, *T. polium*, *L. dentata*, and *L. stoechas* were collected from different sites within the Tafoughalt forest, Berkane Province (eastern Morocco), in May 2021. The species were collected from various locations (Figure 8). Taxonomic identification was performed by Dr. Kaoutar Aboukhalil and Dr. Abdesselam Maatougui based on diagnostic morphological characters using standard botanical keys and regional floras (e.g., *Flore pratique du Maroc*). The accepted scientific names were verified against authoritative taxonomic databases, including Plants of the World Online (POWO) from the Royal Botanic Gardens, Kew. Voucher specimens are deposited at the National Institute of Agronomic Research (INRA, Oujda, Morocco) and are available upon request. The leaves were weighed and air-dried at room temperature under controlled conditions, avoiding light and moisture. EO content was determined based on the weight of air-dried leaves.

### 3.2. Extraction of Essential Oil Using Hydro-Distillation (HD)

To isolate the EOs, all air-dried samples (100 g each) were subjected to Hydro-distillation for 3 h using a Clevenger-type apparatus. The resulting oils were then dried using sodium sulfate (Na_2_SO_4_) and stored in tightly closed, dark vials at 4 °C until analysis [16]. All chemicals and reagents were purchased from Sigma-Aldrich (St. Louis, MO, USA), unless otherwise stated. EO yield was calculated on a dry-weight basis as a percentage (*w*/*w*) using the mass of EO obtained after hydrodistillation relative to the mass of air-dried leaves (Equation (1)):

(1) EO yield (%) = (mass of EO (g)/mass of air − dried leaves (g)) × 100

### 3.3. Qualitative Analysis Using Gas Chromatography-Mass Spectrometry (GC-MS)

A Shimadzu GC-2010 gas chromatograph (Kyoto, Japan) was used to analyses the extracted EOs. Equipped with an RTX-5 capillary column (5% diphenyl, 95% dimethylpolysiloxane, dimensions: 30 × 0.25 mm, film thickness: 0.25 µm; Restek Corporation, Bellefonte, PA, USA)) and coupled with a mass spectrometer (QP2010-MS; Shimadzu, Kyoto, Japan). Helium was used as the carrier gas, maintained at a constant pressure of 100 KPa. The oven temperature was first set to 50 °C for 1 min. Subsequently, the temperature was increased at a rate of 10 °C per minute until it reached 250 °C, which was then maintained for 1 min. For both qualitative and semi-quantitative analysis, a 1 µL sample (prepared in hexane at a concentration of 50 mg/g) was injected in split mode, with a split ratio of 50–80. The GC-MS system operated in scan mode. The chemical composition of the samples was identified by comparing their mass spectra with data from the National Institute of Standards and Technology (NIST147), Wiley, and Adams (4th Edition) mass spectral libraries. Data acquisition and processing were carried out using LabSolutions software (version 2.5; Shimadzu, Kyoto, Japan).

GC-MS profiling was performed as a single analytical run per EO sample by the institutional analytical platform at the Faculty of Science (Oujda, Morocco). While triplicate injections are generally preferred to quantify analytical precision, the single-run approach was dictated by the standard operating procedures of the centralized platform. To ensure the reliability of the chemical profiling despite this limitation, compound identification was rigorously confirmed using a dual-criteria approach, matching experimental retention indices (RI_EXP_) with literature values (RI_LIT_) and mass spectral libraries. Furthermore, the high resolution of the obtained total ion chromatograms (TICs) (Figure 2) and the focus on major constituents (representing the bulk of the oils’ bioactivity) provide a robust basis for the subsequent antioxidant and genotoxic evaluations.

### 3.4. Antioxidant Activity of EOs

#### 3.4.1. DPPH Radical Scavenging Test

The antioxidant activity of the EOs was assessed using the DPPH radical scavenging assay. A DPPH solution was prepared by dissolving 2 mg of DPPH in 50 mL of methanol. Each EO was serially diluted in methanol to obtain a concentration range selected to bracket the IC_50_ (typically 0.25–20 mg/mL, depending on the EO). For the assay, 20 µL of each EO dilution was mixed with 180 µL of the DPPH solution in a 96-well plate (final volume 200 µL). The reaction mixtures were incubated for 30 min at room temperature in the dark, and absorbance was measured at 517 nm using a UV–Vis spectrophotometer (Perkin-Elmer Model Lambda 25, Shelton, WA, USA). Methanol was used as the blank, and a DPPH solution mixed with methanol was used as the negative control. BHT was tested under the same conditions as a positive control. Each condition was analyzed in triplicate (*n* = 3), and results are expressed as mean ± SD. The percentage inhibition (*I*%) was calculated using Equation (2). The IC_50_ values were determined from the dose–response curve by plotting *I*% versus EO concentration and calculating the concentration providing 50% inhibition using nonlinear regression (sigmoidal dose–response) [82].

(2) $$I\%=\frac{ABS_{control}-ABS_{test}}{ABS_{control}}\times 100$$

where *ABS_control_* and *ABS_test_* are the control and sample absorbances at 517 nm, respectively.

#### 3.4.2. β-Carotene Bleaching Assay

We used the β-carotene bleaching test to assess the EO’s potential for antioxidants. Initially, 2 mg of β-carotene were dissolved in 1 milliliter of chloroform to create a β-carotene solution. Next, this mixture was put into a flask with 200 mg of Tween 80 and 2 mg of linoleic acid. After evaporating the chloroform under vacuum (at 40 °C), we introduced 100 mL of oxygen-saturated distilled water with vigorous stirring. Next, 180 µL of the β-carotene solution was transferred to a tube, and 20 µL of the EO at different concentrations were added. The resulting emulsion was incubated in the dark at 50 °C for 2 h. Absorbance measurements were taken at 490 nm both before and after the incubation process. To determine the relative antioxidant activity (*I*%), we used the following formula (Equation (3)):

(3) $$I\%=\frac{A_{sample}(2\;h)-A_{control}(2\;h)}{A_{sample}(0\;h)-A_{control}(2\;h)}\times 100$$

where *I*%, *A_sample_* (2 h), *A_sample_* (0 h) and *A_control_* (2 h) are the percentage of inhibition, the absorbance of the sample after 2 h of incubation, the absorbance of the sample before incubation, and the absorbance of the negative control after incubation, respectively.

#### 3.4.3. FRAP Test

The antioxidant capacity of the EO was evaluated using the Ferric Reducing Antioxidant Power (FRAP) assay. A 1% potassium ferricyanide (K_3_Fe(CN)_6_) solution and 2.5 mL of phosphate buffer (0.2 M, pH 6.6) were combined with 1 mL of each sample. Then the mixture was incubated for 20 min at 50 °C. To stop the reaction, 2.5 mL of 10% trichloroacetic acid was added, followed by centrifugation at 3000 rpm (650 g) for 30 min. The resulting supernatant (2.5 mL) was mixed with 250 µL of distilled water and 0.5 mL of a 0.1% FeCl_3_ solution. Vitamin C was used as the reference standard. Finally, we measured the absorbance at 700 nm using a blank containing methanol [83].

### 3.5. Genotoxic Effect of EO

#### 3.5.1. Blood Sampling and Treatment of Cells

Whole blood samples were collected from male Wistar rats under pentobarbital anesthesia by retro-orbital sampling into heparinized tubes. All experimental procedures involving animals were conducted in accordance with the fundamental ethical principles for animal research, aligning with international guidelines, including the Declaration of Helsinki. The study protocol was reviewed and approved by the Vice-Dean of Scientific Research at the Faculty of Sciences, Mohammed First University, Oujda, Morocco (Trial Registration Reference: 07/25-LBBES).

Because the genotoxicity assessment was performed on freshly collected whole blood/leukocytes (ex vivo), no established cell lines were used, and therefore no cell-line supplier applies. In this study, blood from one healthy rat donor (*n* = 1) was used as the biological source, and independent aliquots from this single sample were processed under all treatment conditions to ensure consistent baseline levels of DNA integrity across the experimental groups. The blood was mixed with 2 mL of Ca^2+^ and Mg^2+^ free PBS solution (137 mM NaCl, 2.7 mM KCl, 10 mM Na_2_HPO_4_, 1.76 mM KH_2_PO_4_ and pH 7.4) for every 2 mL of blood. The blood samples were carefully diluted and exposed to the test substances. The EO, which had been mixed with dimethyl sulfoxide (DMSO), underwent further dilution using phosphate-buffered saline (PBS) to achieve concentrations of 2.5, 5, and 10 µg/mL. The blood cell suspension (10 µL) was kept with the EO for 2 h at 37 °C. The remaining amount of DMSO in the media was less than 0.2%, while the same amount of solvent was used for the negative control. Hydrogen peroxide (H_2_O_2_) was the positive control at a concentration of 250 µmol/L.

#### 3.5.2. Comet Assay

The comet assay was performed with some small changes from the method by Ouahhoud et al. [84]. For the alkaline comet assay, the suspension was spun for 10 min at 4500 rpm. Remove the supernatant and mix the leukocyte-containing pellet with 1 mL of PBS. This step was done three times. After the final spin, the pellet was dissolved in low-melting agarose (LMP) (0.5% *w*/*v* in PBS) and put on a slide with normal-melting agarose (NMP) (1.5% *w*/*v*). Cells embedded in agarose were disrupted using a solution containing 2.5 M NaCl, 100 mM Na2EDTA, 20 mM Tris, 300 mM NaOH, 1% N-lauroylsarcosine sodium, 10% DMSO, and 1% Triton X-100. This disruption process lasted for 5 min, followed by incubation in darkness at 4 °C for 60 min. Finally, we thoroughly rinsed the slides with bi-distilled water. A horizontal gel electrophoresis apparatus was used to hold the slides, which were covered with an electrophoresis solution (pH 13; 300 mM NaOH, 1 mM Na2EDTA). The DNA was unwound for 20 min at 300 mA and 25 V. Migration was carried out for 20 min. The electrophoresis solution was at 4 °C. After electrophoresis and migration, each slide was soaked in neutralization buffer (400 mM Tris-HCl, pH 7.5) for 5 min, three times. Comets were stained with ethidium bromide and observed as described by Singh et al. [85].

#### 3.5.3. Microscopic Observation

The microscopic observation was applied following the methodology described by Singh et al. [85] to quantify DNA damage in cells. A fluorescence microscope (ZOE Fluorescent Imager, Bio-Rad Laboratories, Marnes-la-Coquette, France) was used to view and capture images of the slides stained with ethidium bromide. The red channel that emits and excites light at 556/20 nm and 615/61 nm was used to visualize the DNA. The DNA damage was quantified using Comet Assay IV software (version 4.3, Perceptive Instruments Ltd., Haverhill, UK) to measure parameters associated with DNA lesions [18]. For the animal donor and each treatment condition, slides were prepared in duplicate (technical replicates). Fifty nucleoids were randomly analyzed per slide (100 cells per donor per condition), using Comet Assay IV software.

### 3.6. Analytical Statistics

GraphPad Prism 5.0 statistical software was used to determine the mean values and standard deviations for genotoxicity assessment and antioxidant activity. One-way ANOVA was used to analyze the data. *p*-values below or equal to 0.05 indicated statistically significant results, while lower *p*-values indicated higher levels of significance. Tukey’s honest significant test was applied to examine the differences among treatment groups further. This post hoc test enabled a thorough comparison of multiple treatment groups, identifying any significant differences among them.

## 4. Conclusions

This study examined the antioxidant potential and genotoxic profile of EOs from four Lamiaceae species (*L. dentata*, *L. stoechas*, *T. polium*, and *Z. hispanica*) harvested in eastern Morocco. Overall, the EOs showed measurable antioxidant capacity in the DPPH, β-carotene bleaching, and FRAP assays, with *L. stoechas* and *L. dentata* exhibiting the strongest performance relative to the reference antioxidants. However, the alkaline Comet assay revealed a clear dose-dependent safety concern: *L. dentata*, *Z. hispanica*, and *T. polium* induced significant DNA damage in leukocytes across the tested concentrations, whereas only *L. stoechas* at 2.5 µg/mL showed the lowest genotoxic impact within our experimental range, making it the most promising candidate for cautious further development. Taken together, these findings support potential applications of these EOs as natural antioxidant ingredients in cosmetics/personal-care formulations, as candidates for pharmaceutical research aimed at modulating oxidative stress, and in food-related contexts (e.g., as natural antioxidants/flavoring) only under appropriate regulatory and safety constraints. In practice, any preliminary consumer use should remain at low concentrations; for dermal products, indicative ranges are typically ~0.1–1.0% for facial/sensitive leave-on products and ~1–2% for general leave-on body products (up to ~3% for rinse-off products), with final levels adjusted according to the specific EO/constituent profile and not exceeding applicable safety limits for the relevant product category. Further studies are required to confirm safety and establish robust recommended doses, including (i) expanded cytotoxicity/viability testing, (ii) complementary genotoxicity assays (e.g., micronucleus/Ames) and in vivo toxicology to derive NOAEL-based exposure limits, (iii) mechanistic studies linking composition to oxidative/genotoxic pathways, and (iv) formulation, stability, and interaction studies (synergy/antagonism) to support safe and standardized applications.

## Data Availability

The data presented in this study are available in the article and Supplementary Materials. Additional raw data supporting the findings are available from the corresponding authors upon reasonable request.

## Supplementary Materials

The following supporting information can be downloaded at https://www.mdpi.com/article/10.3390/molecules31030400/s1, Table S1: Major constituents (area %) identified by GC–MS in essential oils from *Ziziphora hispanica*, *Teucrium polium*, *Lavandula stoechas*, and *Lavandula dentata* (Eastern Morocco); and Table S2: Comet assay in rat leukocytes: descriptive statistics (mean ± SD) and Holm-adjusted *p*-values versus the negative control.
