# Supplementary material for: Chemical Composition, Antioxidant Potential, and Genotoxic Safety of Lamiaceae Essential Oils from Eastern Morocco: A Multimethod Evaluation
Source: Molecules. 2026 Jan 23;31(3):400. doi: 10.3390/molecules31030400 (PMC12898240; doi:10.3390/molecules31030400)
Supplement: Supplementary file 1 [file molecules-31-00400-s001.zip › molecules-4046809-supplementary.pdf]

Supplementary File

Chemical Composition, Antioxidant Potential, and Genotoxic Safety of Lamiaceae Essential Oils from Eastern Morocco: A Multimethod Evaluation

Abderrahman Makaoui [1,2], Abdelmonaem Talhaoui [2], Kaoutar Aboukhalid [3], Rachid Sabbahi [4,*], Sabir Ouahhoud [5], Sanae Baddaoui [1], Abdessadek Essadek [1], Abdesselam Maatougui [3], Ennouamane Saalaoui [1] and Mounsef Neffa [1,*]

[1] Laboratory of Bioresources, Biotechnology, Ethnopharmacology and Health, Faculty of Sciences, Mohammed First University, BV Mohammed VI BP 717, Oujda 60000, Morocco; abderrahman.makaoui@etu.uae.ac.ma (A.M.); sanaebaddaoui@gmail.com (S.B.); a.essadek@ump.ac.ma (A.E.); e.saalaoui@ump.ac.ma (E.S.)

[2] Physical Chemistry of Natural Substances and Process Team, Laboratory of Applied Chemistry and Environment (LCAE-CPSUNAP), Department of Chemistry, Faculty of Sciences, Mohammed First University ,BV Mohammed VI BP 717, Oujda 60000, Morocco; talhaouiabdelmonaem@gmail.com

[3] National Institute of Agronomic Research, CRRA Oujda, 10 Bd Mohamed VI, B.P. 428, Oujda 6000, Morocco; k.aboukhalid@yahoo.com (K.A.); abdesselam.maatougui@inra.ma (A.M.)

[4] Research Team in Science and Technology, Higher School of Technology of Laayoune, Ibn Zohr University, P.O. Box 3007, Laayoune, Morocco

[5] Laboratory of Health Sciences, Artificial Intelligence, and Applied Nanotechnology, Faculty of Medicine and Pharmacy, University Sultan Moulay Slimane, Beni Mellal 23000, Morocco; s.ouahhoud@ump.ac.ma

* Correspondence: r.sabbahi@uiz.ac.ma (R.S.); m.neffa@ump.ac.ma (M.N.)

Table S1. Major constituents (area %) identified by GC–MS in essential oils from *Ziziphora hispanica, Teucrium polium, Lavandula stoechas, and Lavandula dentata* (Eastern Morocco)

Compound	RI_{EXP}	RI_{LIT}	RT	Area (%)					Reference
				Ziziphora hispanica	*Teucrium polium*	*Lavandula stoechas*	*Lavandul a dentata*	Method of identification	
∝-Pinene	934	939	5.217	1.20	2.94	0.43	12.09	RI, MS	[35]
βMyrcene-	979	988	6.110	-	1.70	-	15.82	RI, MS	[34]
Eucalyptol	1032	1016	6.855	40.08	1.66	25.04	-	RI, MS	[37]
Fenchone	1092	1094	7.832	-	-	30.69	-	RI, MS	[36]
Camphor	1143	1146	8.802	-	-	11.77	-	RI, MS	[35]
Menthol	1161	1167	9.136	10.77	-	-	-	RI, MS	[46]
d-p-Menth-4(8)-en-3-one	1233	1233	10.287	12.74	-	-	-	RI, MS	[49]
(R)-(+)-3-Methyladipic acid	1294	1315	11.407	10.37	-	-	-	RI, MS	[57]
Caryophyllene	1420	1413	13.014	-	-	-	12.10	RI, MS	[63]
Germacrene D	1480	1481	13.849	-	-	-	8.96	RI, MS	[38]
Thujopsene	1497	1497	14.052	-	11.25	-	-	RI, MS	[69]
T-Cadinol	1640	1644	15.925	-	8.84	-	-	RI, MS	[38]
∝-Bisabolol	1680	1682	16.362	-	8.01	-	-	RI, MS	[39]

RIExp = experimental retention index; RILit = literature retention index from the Adams, Wiley and NIST libraries, supported by bibliographic references; and RT= retention time.

Table S2. Comet assay in rat leukocytes: descriptive statistics (mean ± SD) and Holm-adjusted p-values versus the negative control

Label	Group	Dose	Tail moment (mean ± SD)	p_adj vs NC	Tail intensity (mean ± SD)	p_adj vs NC	Tail length (mean ± SD)	p_adj vs NC
A	Negative control	-	4.05 ± 7.71	–	9.86 ± 15.2	–	65.8 ± 25.8	–
B	*Lavandula stoechas*	2.5 µg/mL	11.8 ± 12.7	<0.0001 (****)	26.5 ± 22.5	<0.0001 (****)	82.5 ± 24.7	0.00537 (**)
C	*Lavandula stoechas*	5 µg/mL	15.1 ± 12.7	<0.0001 (****)	32 ± 19	<0.0001 (****)	95.4 ± 19.7	<0.0001 (****)
D	*Lavandula stoechas*	10 µg/mL	16.6 ± 16.6	<0.0001 (****)	33.3 ± 23	<0.0001 (****)	97.7 ± 32.2	<0.0001 (****)
E	*Lavandula dentata*	2.5 µg/mL	19.2 ± 15.3	<0.0001 (****)	38.6 ± 19.9	<0.0001 (****)	104 ± 27.4	<0.0001 (****)
F	*Lavandula dentata*	5 µg/mL	23.3 ± 17.1	<0.0001 (****)	44.8 ± 21.9	<0.0001 (****)	108 ± 25.8	<0.0001 (****)
G	*Lavandula dentata*	10 µg/mL	27.5 ± 25.7	<0.0001 (****)	45.1 ± 26.1	<0.0001 (****)	128 ± 47.4	<0.0001 (****)
H	*Ziziphora hispanica*	2.5 µg/mL	16.7 ± 11	<0.0001 (****)	37.5 ± 18.6	<0.0001 (****)	94.2 ± 19	<0.0001 (****)
I	*Ziziphora hispanica*	5 µg/mL	20 ± 12.7	<0.0001 (****)	39.9 ± 20.8	<0.0001 (****)	108 ± 23.4	<0.0001 (****)
J	*Ziziphora hispanica*	10 µg/mL	25.1 ± 23.7	<0.0001 (****)	43.9 ± 26	<0.0001 (****)	120 ± 37.7	<0.0001 (****)
K	*Teucrium polium*	2.5 µg/mL	18.8 ± 10.6	<0.0001 (****)	38.6 ± 19.8	<0.0001 (****)	106 ± 21.3	<0.0001 (****)
L	*Teucrium polium*	5 µg/mL	22.1 ± 12.5	<0.0001 (****)	44.1 ± 19.5	<0.0001 (****)	115 ± 22.7	<0.0001 (****)
M	*Teucrium polium*	10 µg/mL	23 ± 14.4	<0.0001 (****)	46.2 ± 23.5	<0.0001 (****)	114 ± 29.7	<0.0001 (****)
N	Positive control (H2O2)	250 µM	19.5 ± 28.4	<0.0001 (****)	31.9 ± 44.3	<0.0001 (****)	71.8 ± 39.8	0.00779 (**)

*Notes: p_adj values were obtained using a Kruskal-Wallis test followed by Dunn's multiple comparisons versus the negative control, with Holm correction for multiple testing. Significance codes: **** p ≤ 0.0001; *** p ≤ 0.001; ** p ≤ 0.01; * p ≤ 0.05; ns p > 0.05.*
